# The Impact of Episodic Memory on Decision-Making in Aging: Scenarios from Everyday Life Situations

**DOI:** 10.3390/brainsci14100998

**Published:** 2024-09-30

**Authors:** Fanny Gaubert, Céline Borg, Hélène Saint-Martin, Stéphanie de Chalvron, Hanna Chainay

**Affiliations:** 1 Laboratoire d’Etude des Mécanismes Cognitifs, Université Lumière Lyon 2, 69007 Lyon, France; fanny-nathalie.gaubert@orange.fr (F.G.); ln.saintm@gmail.com (H.S.-M.); 2 Centre Hospitalier Universitaire de Saint-Etienne, Hôpital Nord, 42270 Saint-Priest-en-Jarez, France; celine.borg@chu-st-etienne.fr; 3 Unité de Recherche Confluence EA 1598, Campus Carnot Institut Catholique de Lyon, 69002 Lyon, France; 4 Laboratoire de Psychologie et Neurocognition, Université Grenoble Alpes, 38400 Saint-Martin-d’Hères, France; 5 SBT Human(s) Matter, 69002 Lyon, France; s.dechalvron@humansmatter.co

**Keywords:** decision-making under risk and under ambiguity, everyday life scenarios, memory, recollection, familiarity

## Abstract

**Background:** Decision-making is a highly complex process that depends on numerous cognitive functions, such as episodic memory. It is also influenced by aging. However, how changes in episodic memory with age contribute to changes in decision-making is not clear yet. **Objective:** This work aimed to examine the role of two memory processes, recollection and familiarity, in decision-making in ageing. **Method:** Thirty young adults and 30 older adults performed two episodic memory tasks: recognition, which allowed for the measurement of recollection and familiarity, and recall, which allowed for the measurement of recollection. In both tasks, they first viewed a series of pictures and then were asked to recognise or recall them respectively. They also performed an original scenario task based on situations inspired by everyday life, evaluating decision-making under conditions of either risk or ambiguity. In this task, participants were presented with short descriptions of situations requiring a decision and had to choose between two given options. **Results:** Lower performances was observed in recall and recognition tasks in older than in young adults. In the scenarios task, young adults sought significantly more risk and ambiguity than older adults. In both young and older adults, recollection and familiarity processes were involved differently in decision-making. The former is more involved in decision-making under ambiguity, and the latter in decision-making under risk. **Conclusions:** The results suggest that decision-making changes with age, but that the involvement of the episodic memory, familiarity and recollection processes, does not appear to vary with age.

## 1. Introduction

### 1.1. Changes in Decision-Making during Ageing

Decision-making is a highly complex process that depends on a large number of factors and changes with age. Modifications in the way older adults make decisions have been identified in both decision-making under risk (i.e., the possible outcomes and their probability of occurrence can be estimated) and decision-making under ambiguity (i.e., the possible outcomes and their probability of occurrence remain uncertain) [1,2,3].

Under ambiguity, there is a general consensus that a modification occurs in late adulthood. Most of the time, this change is shown with the Iowa Gambling Task (IGT) (e.g., [4,5,6,7,8,9]). Studies using other tasks, such as the Baloon Analogue Risk Task (BART), have also been conducted and have shown age-related changes ([10] for a review). However, the IGT task is controversial as its ecological validity is questioned, and the nature of the decision that is tested strongly depends on the phase of the task [11]. Furthermore, as pointed out in the review by [10], contrary results were obtained with IGT and BART concerning decision-making under ambiguity in older adults. They were more risk averse in BART but took more risks and made less advantageous decisions in IGT. Interestingly, a study by [7] used IGT and developed another task consisting of different scenarios under ambiguity (and under risk), all based on everyday life situations. The IGT did not reveal any significant differences in decision-making between young and older adults. On the contrary, under ambiguity conditions in the scenarios task, a significant effect of age was shown, with older adults selecting significantly fewer ambiguous options than young adults.

Under risk, the findings have been mixed, with some studies indicating similar decision-making performance in young and older adults [9,12,13], whereas other studies have pointed to age-related changes [7,14]. For example, as in the under-ambiguity condition in the scenarios task, ref. [7] showed that in the under-risk condition, older adults selected significantly fewer risky options than young adults. It is thought that the range of different tasks used is responsible for this heterogeneity [15]. The decision-making tasks employed in studies may exhibit variations, including differences in ecological validity.

In the current research, which explores factors contributing to age-related changes in decision-making, we focused on scenarios rather than the IGT task. This decision was based on the results of our previous study [7], which showed no differences between young and older adults with the IGT task but did find differences in the scenarios task under both ambiguity and under risk. To better reflect real-life decision-making, we decided to use only scenarios as a task. As [16] have suggested that young and older adults may display distinct decision-making patterns based on the level of certainty within situations, our scenarios included probabilities for various options to assess this aspect more effectively.

### 1.2. The Role of Episodic Memory in Decision Making

There is a growing awareness among researchers that episodic memory plays a crucial role in decision-making, along with other cognitive functions. Indeed, individuals can assess different actions based on their expected rewards, gains or losses. Episodic memories can contribute to this process by providing a broader context for evaluating actions. By recalling past experiences, individuals can assess the long-term consequences of their choices and avoid repeating negative outcomes. This ability to simulate scenarios based on episodic memories allows for more informed and sophisticated decision-making.

Although recent research has demonstrated that decision-making involves episodic memory [17,18,19,20,21,22,23,24,25], the specific mechanisms underlying these processes remain uncertain. Many decisions in our daily lives involve choosing between various options. Since the likelihood of the outcomes is often unknown, we frequently rely on memories of similar past experiences to guide our choices [26,27]. Successful decision-making requires individuals to combine insights from past experiences with current environmental demands. Research indicates that when faced with extensive information, focusing on key details can enhance decision-making efficiency [28,29]. As older adults may experience changes in episodic memory, this could potentially influence their decision-making process.

### 1.3. Impact of Memory Changes during Ageing on Decision-Making

From a behavioural perspective, ageing affects episodic memory by reducing the precision of memory retrieval [30] and making it more challenging to bind various components of an episode into a coherent whole [31]. Moreover, some results showed that distinct genetic profiles underlie specific mental processes of human episodic memory [32,33]. Different tasks can be used to assess episodic memory: recall (i.e., free or cued) or recognition (i.e., simple or with a Remember/Know paradigm). Recognition tasks involve retrieving previously encoded information based on current cues, with recognition being an automatic process driven by familiarity, although recollection may also play a role. In contrast, free or cued recall tasks are not based on information currently present. Recall requires an active search through memory relying on recollection for controlled access to information. The Remember/Know (R/K) paradigm is commonly used to study the involvement of recollection and familiarity in recognition tasks [34]. Participants have to indicate whether their recognition responses are based on the recollection of specific details (remembering) or on a general sense of familiarity without specific details (knowing). However, the R/K paradigm has been criticised for certain procedural limitations, leading to the development of various modifications [35]. To better determine whether recollection or familiarity was used to recognise items, some researchers used a modified R/K procedure and explicitly asked the participants about contextual or item-specific details [36,37,38].

The ageing process typically results in impairments in free recall [39] and recollection performances [40,41,42,43], although some studies did not report age-related differences in recollection performance (e.g., [44,45]). However, there is no clear consensus regarding the familiarity process. While most studies have found that familiarity is preserved in older adults [40,41,42], some studies have reported a decline with age [45,46], with [43] noting that the results depend on the tasks used. For instance, studies have shown that older adults may struggle with item recognition when the lures are similar to the targets [47]. This has led researchers to think that there is a familiarity bias in ageing, at least when stimuli are distinctive. Very few studies have explicitly examined the involvement of recollection and familiarity in decision-making, either in young or older adults. In addition, [22] lower levels of risk-taking were found following the induction of general impressions (i.e., familiarity process) than following the induction of episodic specificity (i.e., recollection process). Therefore, a decline in the recollection process, such as in ageing, would contribute to a more conservative approach to decision-making.

### 1.4. Objective

Several studies have suggested that decision-making may rely on the integrity of long-term memory, whether risk or ambiguity. In addition, both decision-making and episodic memory have been shown to change with age. However, too few studies have examined the link between the two. Thus, the present study aimed to examine the relationship between decision-making under ambiguity and risk and episodic memory, especially its two processes, recollection and familiarity, using tasks based on everyday situations. To do so, recall and recognition tasks were used.

As it was found that young adults choose risky and ambiguous options more frequently than older adults [7], we therefore expected to observe the same pattern of results. When people confront uncertain situations, they may also draw on their episodic experiences to help inform their choices. Reduced recollection with ageing should, therefore, destabilise their decision-making, and this should be even more so under ambiguity when the probabilities are very unclear. In other words, lower recollection scores could predict a reduced tendency to choose scenarios with a high degree of uncertainty in a decision-making task. As age particularly affects the recollection process, these results are particularly expected in older adults. In other words, with regard to the involvement of memory in decision-making, our hypothesis is that, unlike young adults, older adults rely more on familiarity processes than recollection processes to make their decisions.

## 2. Materials and Methods

### 2.1. Transparency and Openness

Data were analysed using JASP (version 0.14.0.0, JASP Team, University of Amsterdam, Amsterdam, The Netherleands, 2022) and SPSS for regression analyses. The regression analysis models are provided in Supplemental Material. This study’s design and its analysis were not pre-registered. The scenarios task and the data of the present study are available at: https://osf.io/j4wzr/?view_only=6ad2e97e00c74e9991d26cda97fe7330 (accessed on 22 August 2024).

### 2.2. Participants 

Two groups of participants were included in the study: a group of 30 older adults (17 women and 13 men) aged between 60 and 83 years (M = 68.9, SD = 6.2) and a group of 30 young adults (17 women and 13 men) aged between 18 and 30 years (M = 24.4, SD = 3.5). The young participants were recruited through announcements in lectures and tutorials, on the EMC laboratory website and through other laboratory communication channels. The older participants were recruited via a list of older volunteers from the EMC laboratory and through advertisements in associations, senior citizens’ clubs, town halls in Lyon and Bron and the website of the Université Tous Ages at the Lyon 2 University. The participants did not receive any remuneration or reward for their participation in this study.

The proportion of men and women was identical. Both groups had comparable levels of education (U = 397.5, *p* = 0.215, d = −0.46). 

Our target sample size was determined for the decision-making task using a *priori* power analysis performed in G*Power [48], using the value of η^2^_p_ = 0.103 for the under-risk scenarios task that had been obtained in our previous study [7]. Our study design for the under-risk task with one between-participants factor (older and young adults groups) and one repeated factor, context (gain and loss), was able to achieve 80% power at α = 0.001 with 22 participants per group. To further strengthen our study, we decided to include 30 participants per group. We chose this task as the reference because the calculation based on the under-ambiguity scenarios gave a lower sample size (10 participants per group).

The older participants underwent a brief neuropsychological assessment consisting of the Mini-Mental State Examination (MMSE), which indicated a preserved global mental status (M = 28.8, SD = 1.3) [49,50], and the brief frontal efficiency battery (Batterie Rapide d’Efficience Frontale, BREF) (M = 16.8, SD = 1.7) [51]. They also underwent the Revised Observed Tasks of Daily Living (OTDL-R) [52], which assesses the level of autonomy in using everyday life objects (e.g., the participants were asked to use the phone, to take medicine from the pillbox, etc.; all the objects necessary to produce the behaviour were provided to the participants). Due to time constraints, we had to reduce this test and therefore used only three items from the healthcare field, one from the communication field, and two from the finance field.

All the participants completed the South Oaks Gambling Screen (SOGS) [53], i.e., an instrument designed to identify pathological gamblers. None of them had a pathological gambling profile. The older adults also completed the Hospital Anxiety and Depression Scale (HADS): anxiety score (M = 7.1, SD = 3.0) and depression score (M = 3.3, SD = 2.3). The young adults completed the Spielberg State–Trait Anxiety Inventory (STAI): state anxiety score (M = 40.5, SD = 13.2) and trait anxiety score (M = 42.7, SD = 10.3). In addition, before the decision-making and episodic memory tasks, the participants’ mood was assessed with the Brief Mood Introspection Scale (BMIS) [54]. Two scores were taken into account: Pleasant/Unpleasant (before the decision-making task, for older adults M = 55.8, SD = 6.4 and for young adults M = 47.6, SD = 6.3; and before the episodic memory task, for older adults M = 56, SD = 6.1 and for young adults M = 46.4, SD = 7.2) and Aroused/Calm (before the decision-making task, for older adults M = 29.3 SD = 2.9 and for young adults M = 27.2, SD = 3.7; and before the episodic memory task, for older adults M = 28.6, SD = 3.0 and for young adults M = 26.3, SD = 3.4). The differences between older and young adults for BMIS scales were statistically significant for both sessions (*p* < 0.05), with older adults being in a more pleasant mood and being more aroused.

None of the participants scored outside the norm in any of the neuropsychological tests. None of the participants was excluded from the study.

The study was approved as a part of a larger project entitled ‘Decision-making deficits in patients with Alzheimer’s Disease: functions involved and the day-to-day consequences’ by the ethical committee (Comité de Protection des Personnes Ile de France XI) and was assigned the number 19031-57438.

### 2.3. Experimental Tasks

All the tasks performed by the older adults were programmed and run on E-Prime 2.0 software (Psychology Software Tools Inc., Pittsburgh, PA, USA) on a Dell laptop with azerty keyboard. Because of the pandemic context, all the tasks performed by the young adults were programmed and run on Psytoolkit [55,56].

Each task began with instructions, an example to illustrate them, and practice trials. The instructions were written on the screen and verbally given by the experimenter to the older adults, who performed the task in the experimenter’s presence. Each participant completed all tasks and experimental conditions.

#### 2.3.1. Decision-Making Assessment

The Scenarios Task was designed for the purposes of an earlier study investigating the links between decision-making, working memory, and executive functions. The description given here is the same as that presented in [7,57].

This experimental task was developed from the protocols of two previous studies and included two subtasks assessing decision-making, one under ambiguity and the other under risk [58,59].

**Figure 1:**
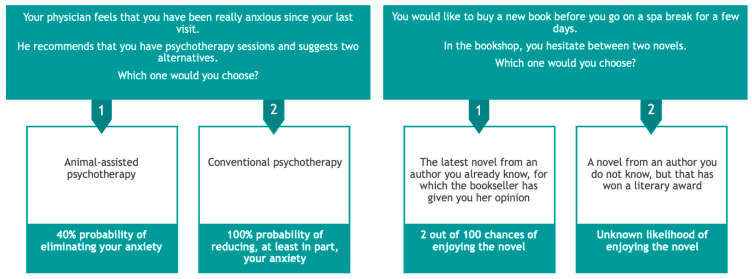
Example of scenarios: on the left, under risk—in this example, the scenario involves a small gain (partially reducing anxiety) versus a larger gain (eliminating anxiety), and on the right, under ambiguity—in this example, the scenario involves an unambiguous small gain versus an uncertain outcome.

Thirty-six brief scenarios related to everyday life situations were proposed in each subtask (see Figure 1 for examples). For each scenario, two or three sentences presented the context at the top of the screen and below them, two possible options were proposed in boxes labelled “1” (on the left) or “2” (on the right). Each scenario was evaluated by a sample of 49 young adults and 10 older adults on the following two scales: plausibility (0—not at all plausible, 5—totally plausible), familiarity with a situation (0—not at all familiar, 5—totally familiar). In addition, older adults evaluated the intelligibility of the scenarios (0—unintelligible, 5—very understandable). We calculated Cronbach’s alpha separately for each age group. The internal consistency of our scenarios for young adults was good for both scales, 0.946 and 0.911, respectively [60]. For older adults, it was also good for all three scales: 0.948, 0.940 and 0.898.

The participant’s task was to choose their preferred option by pressing the “1” or “2” key on the keyboard. The scenario with its options disappeared after participants had made their choice, and the next scenario appeared. In the risk condition, half the scenarios concerned loss situations and the other half gain situations. The choice was made between a less winning/losing but certain option (with a probability of 100%) and a more winning/losing but less certain option (with a probability of x%). In the ambiguity condition, 4 different winning scenarios were proposed and were repeated with nine different probabilities, giving 36 trials. Participants chose between an option with a known probability (x%) and one with an unknown probability. Under both risk and ambiguity conditions, the probability values for x ranged from 2% to 98% (2, 10, 20, 40, 50, 60, 80, 90 and 98%). Furthermore, in half of the trials, probabilities were expressed as percentages (e.g., 10% chance of arriving on time) and in the other half, they were expressed as frequencies (e.g., 10 chances out of 100). The presentation of the two options and numerical information was balanced from trial to trial. The trial presentation was random in the risk condition and semi-random in the ambiguity condition.

#### 2.3.2. Recognition Task

**Figure 2:**
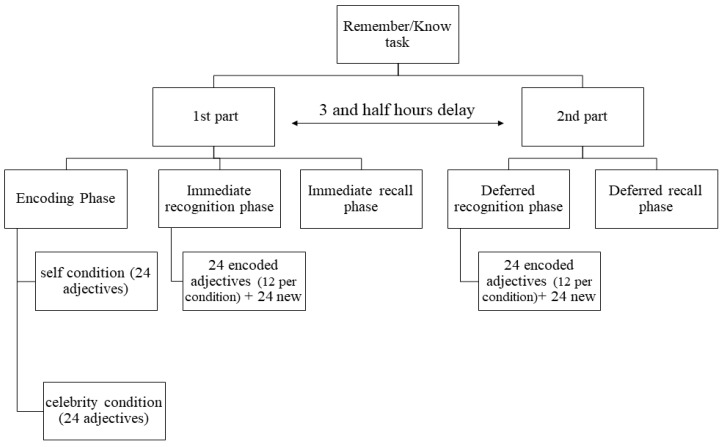
Diagram describing the Remember/Know task procedure.

Episodic memory was measured using a recognition task inspired by the study by [36] in which states of consciousness associated with the retrieval of items, or in other words, the processes used, recollection and familiarity, respectively, were assessed by the recalled details from the encoding context. Ninety-six adjectives were selected and organised into 4 lists of 24 words each. In each list, half of the adjectives had a positive and the other half a negative valence. The four lists were similar in terms of frequency, number of letters and number of syllables. The task was divided into two parts, consisting of three and two phases, respectively (see Figure 2). During the first part, the encoding phase started once the instructions and examples had been presented. Two conditions were presented one after the other: self and celebrity condition. We used these two conditions to ensure deep encoding. In the self-condition, participants had to decide whether the adjectives presented on the screen corresponded to their personality or not. The question “Are you?” was written on the top of the screen, and the adjectives were presented in the centre, one after the other. When the experiment was conducted in the presence of the experimenter (i.e., for the older adults), the participants answered “yes” by pressing the “l” key labelled with a blue sticker and “no” by pressing the “s” key labelled with a red sticker. In remote mode (i.e., for the young adults), the participants answered “yes” by pressing the “2” key and “no” by pressing the “1” key, both on the main keyboard. The next adjective appeared once one of the two answer keys had been pressed. The adjectives, which were written in French, agreed with the participant’s gender (i.e., feminine vs. masculine). In the celebrity condition, the same procedure was followed and differed only in that instead of answering the question “Are you?” the participants had to answer for a celebrity. For the older adults group, the target celebrities were “Catherine Deneuve” and “Michel Sardou”, i.e., a French actress and a French singer. For the young adults group, the celebrities were “Marion Cotillard” and “Jean Dujardin”, i.e., a younger French actress and French actor. Twenty-four adjectives were presented for each condition, meaning that 48 adjectives were encoded in total. Directly after the encoding phase, the participants had to perform a retrieval phase. Forty-eight adjectives were presented one after the other. Of these, 24 had been presented during the encoding phase (i.e., 12 per encoding condition, self and celebrity), and 24 were new. The participants had to answer the question, “Have you already seen this adjective?”. The same response keys as for the encoding phase were used to answer yes or no. If the answer was “no”, the adjective disappeared, and the next one was displayed on the screen. However, if the answer was “yes”, the participant was tested further to determine whether this answer was given on the basis of the familiarity or recollection process. Inspired by studies by [36], we decided to ask participants to answer the following three questions rather than asking them to answer ‘remember’ or ‘know’: (A) What question did this adjective follow?: (1) Are you?, (2) Marion Cotillard (e.g.,) is?, (3) I don’t remember; (B) What did you answer?: (1) yes, (2) no, (3) I don’t remember; and (C) Reading this adjective, did you have: (1) an image or memory in mind, or a particular feeling?, (2) none of these, (3) I don’t remember. If the participants answered C.1., a text area appeared in which the participants were asked to describe their image/memory/feeling. If the participants answered “I don’t know” for the three complementary questions, the recognition was considered based on the familiarity process. If the participants had at least one precise memory of the encoding phase (i.e., the condition, the answer, the image/memory/feeling), the recognition was considered based on the recollection process. Once the recognition list had been completed, a free recall phase started. The participants were instructed to recall as many of the presented words (i.e., from the encoding phase and the new words) in a maximum of five minutes. The older adults spoke the adjectives aloud, and the experimenter wrote them down. The young adults saw a special text area in which they could type the recalled adjectives by themselves. The second part of the task took place three-and-a-half hours later. This started with a delayed recognition phase and used the 24 encoded words (i.e., 12 per condition) not displayed during the immediate retrieval phase, together with 24 new adjectives. The same procedure was followed as in the first recognition phase. Once the 48 adjectives had been presented, a delayed recall phase started. The participants were asked to give the maximum number of adjectives they could remember from both parts of the task taken together.

Eight versions of the task were programmed in order to counterbalance the encoding condition order as well as the lists of encoded adjectives.

### 2.4. Procedure

For all participants, the experiment consisted of two sessions separated by a three-and-a-half-hour break. For health reasons, due to the pandemic, young participants completed the second session, and older adults completed both sessions at home. The young adults came to the laboratory to receive all the instructions for the remote tasks to perform a decision-making task that is not relevant to this study, and on that occasion, they performed the Spielberg State–Trait Anxiety Inventory. After this visit, during the same day, they received two e-mails which they were asked to open in order of their arrival. The first one contained the instructions (i.e., written and spoken) for the recognition task and recall task, as well as the link to the BMIS questionnaire and the two links, respectively, to the first recognition and recall task and to the second recognition and recall task. The experimenter specifically emphasised two points: (1) the need to respect the three-and-a-half-hour break between the two recognition and recall tasks and (2) the need to perform the task in a quiet environment. The second e-mail contained the instructions for the scenarios task, the under-risk and the under-ambiguity condition, as well as the links to the BMIS questionnaire. The participants were asked to respond to the questionnaire and perform the two conditions of the scenarios task in sequence, once again, in a quiet environment.

The older adults performed the tasks at home with the experimenter. During the first session, they performed the neuropsychological examination, followed by the SOGS and the HAD scale. They then performed the first recognition and recall tasks. The second session took place three-and-a-half hours later, starting with the second recognition and recall task, followed by a decision-making task that is not relevant to this study, the OTDL-R and the scenarios task.

In summary, the young and the older adults performed the memory tasks and the scenarios tasks at home. The young adults did this on their own (they received all the instructions they needed to carry out these tasks when they were in the laboratory and received them again by e-mail), and the older adults carried out the tasks with the experimenter accompanying them throughout the experiment.

## 3. Results

### 3.1. Group Comparisons

Before conducting statistical analyses, sphericity (using Mauchly’s test), normality of the distribution (using Shapiro–Wilk), and homogeneity of variance (with Levene’s test) were checked. The Greenhouse–Geisser correction was applied if sphericity was violated, and the Mann–Whitney U test was employed if the normality of distribution was not respected.

#### 3.1.1. Episodic Memory

Episodic memory was assessed by means of recall and recognition tasks in both immediate (i.e., just after the encoding phase) and delayed (i.e., 3 ½ h after the encoding phase) conditions. The recall score corresponded to the number of recalled adjectives from the encoding list. For the recognition task, HITs and FAs for recollection-type and familiarity-type responses were taken into account, and d’ and C indices were calculated for immediate and delayed recognition for each response type. These indices reflect the ability to distinguish older from new items and the decisional strategy (i.e., favouring “yes” answers, which reflected a liberal strategy, vs. “no” answers, which reflected a conservative strategy), respectively [61,62]. The data and results of the statistical analyses are presented in Table 1.

Significant differences were observed for immediate and delayed recall, with older adults recalling fewer items than young adults. In the recognition task, older adults made significantly fewer familiarity-type Hits than young adults, and their d’ index for this type of response was significantly lower than that of young adults. In addition, for this type of response, the C index of older adults was significantly higher than that of young adults. There were no other significant differences between these two groups in the memory tasks.

#### 3.1.2. Scenarios Task

In the statistical analyses, we took into account the number of risky and ambiguous decisions (i.e., under-risk and under-ambiguity tasks, respectively). First, we ran an ANOVA on the total number of risky and ambiguous choices with a group (older adults vs. young adults) as a between-subject factor and condition (under risk vs. under ambiguity) as a within-subject factor. We then ran a separate analysis for each decision-making condition. For the under-risk condition, repeated-measure ANOVA was performed on the number of risky decisions, with the group as between-subject factor, and context (gain vs. loss) and certainty (2%, 10%, 20%, 40, 50, 60, 80, 90 and 98%) as within-subject factors. In addition, another ANOVA was run, with a group (older adults vs young adults) as between-subject factors and context (gain vs. loss) and numerical presentation (percentage vs. frequency) as within-subject factors. For the under-ambiguity condition, the ANOVAs were performed with group and certainty, and group and numerical presentation were also performed. If necessary, the analyses were followed by post-hoc comparisons.

The repeated-measures ANOVA on the number of risky and ambiguous choices revealed significant simple effects of group (F(1, 58) = 28.8, *p* < 0.001, η_p_^2^ = 0.332) and condition (F(1, 58) = 24.1, *p* < 0.001, η_p_^2^ = 0.294). Globally, risky options (mean = 18.6, SD = 3.1) were chosen more frequently than ambiguous options (mean = 15.3, SD = 5.3), and young adults (mean = 18.8, SD = 3.6) more frequently selected risky/ambiguous options than older adults (mean = 14.9, SD = 4.84). The interaction between these two factors was not significant (F(1, 58) = 0.49, *p* = 0.0.48, η_p_^2^ = 0.008).

Under-Risk Condition

The repeated-measures ANOVA on the number of risky choices revealed significant simple effects of group (F(1, 58) = 19.2, *p* < 0.001, η_p_^2^ = 0.248), context (F(1, 58) = 135.6, *p* < 0.001, η_p_^2^ = 0.700) and percentage of certainty (F(6.4, 396.8) = 4.8, *p* < 0.001, η_p_^2^ = 0.076). In general, more risks were taken by the young adults (mean = 10.1, SD = 3.3) than by the older adults (mean = 8.4, SD = 3.5), and more risks were taken in the loss context (mean = 12.1, SD = 2.1) than in the gain context (mean = 6.5, SD = 2.1). Most importantly, the percentage value of the certainty*context*group interaction (F(6, 464) = 16, *p* < 0.001, η_p_^2^ = 0.216) was significant.

To better understand this interaction, we conducted two separate ANOVAs, one for the gain condition and another for the loss condition, with the group being between-subject factor and the percentage of certainty being within-subject factor.

**Figure 3:**
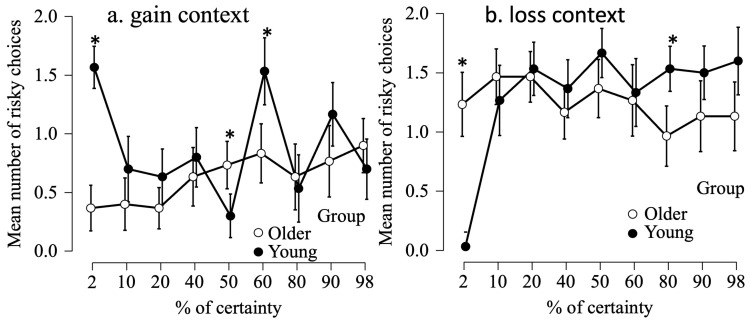
The average number of risky options chosen in gain (**a**) and loss (**b**) scenarios as a function of the certainty percentage of those options. Errors bars indicate standard errors. The stars indicate significant comparisons * *p* < 0.001, except 50% in gain context when *p* is < 0.002.

In the gain context, we observed significant effects of group (F(1, 58) = 19.1, *p* < 0.001, η_p_^2^ = 0.248, percentage of certainty (F(6.5, 379.5) = 4.3, *p* < 0.001, η_p_^2^ = 0.124) and an interaction between these two factors (F(6.5, 379.5) = 8.4, *p* < 0.001, η_p_^2^ = 0.127). After Bonferroni correction, planned comparisons revealed significant differences between young and older adults for 2% (t(58) = −9.34, *p* < 0.001, d = −2.41), 50% (t(58) = 3.17, *p* < 0.002, d = 0.82) and 60% (t(58) = −3.45, *p* < 0.001, d = −0.89) of certainty, with young adults making more risky choices than older adults for 2% and 60% of certainty, and the reverse being the case for 50% of certainty (see Figure 3a).

In a loss context, we observed significant effects of group (F(1, 58) =1.48, *p* = 0.22, η_p_^2^ = 0.025), percentage of certainty (F(1, 464) = 9.0, *p* < 0.001, η_p_^2^ = 0.135) and an interaction between these two factors (F(1, 464) = 9.4, *p* < 0.001, η_p_^2^ = 0.140). After Bonferroni correction, planned comparisons revealed significant differences between young and older adults for 2% (t(58) = 8.75, *p* < 0.001, d = 2.61) and 80% (t(58) = −3.69, *p* < 0.001, d = −0.95) of certainty, with young adults taking less risky choices than older adults at 2% of certainty, and the reverse being true for 80% of certainty (see Figure 3b).

**Figure 4:**
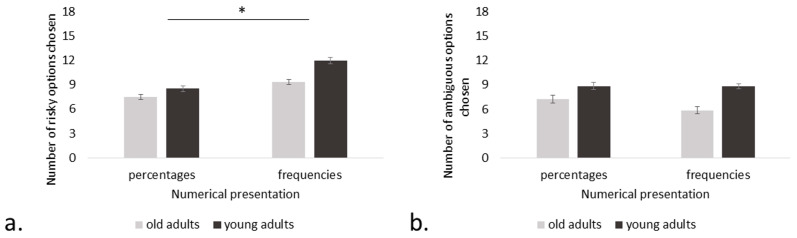
Average selections of risky (**a**) and ambiguous (**b**) options based on numerical presentation (percentages, frequencies). In both figures, the bars represent standard deviation errors, with a maximum of 18 choices for risky and ambiguous options. The star indicates significant comparison, *p* < 0.001.

Finally, the ANOVA, including the numerical information factor (frequency, percentage), showed a significant simple effect of numerical presentation (F(1, 58) = 26.0, *p* < 0.001, η^2^ = 0.214), with more risks being taken when the information was presented in frequencies (see Figure 4a). No numerical presentation*group interaction effect appeared (F(1, 58) = 2.4, *p* = 0.127, η^2^ = 0.020).

Under-Ambiguity Condition

**Figure 5:**
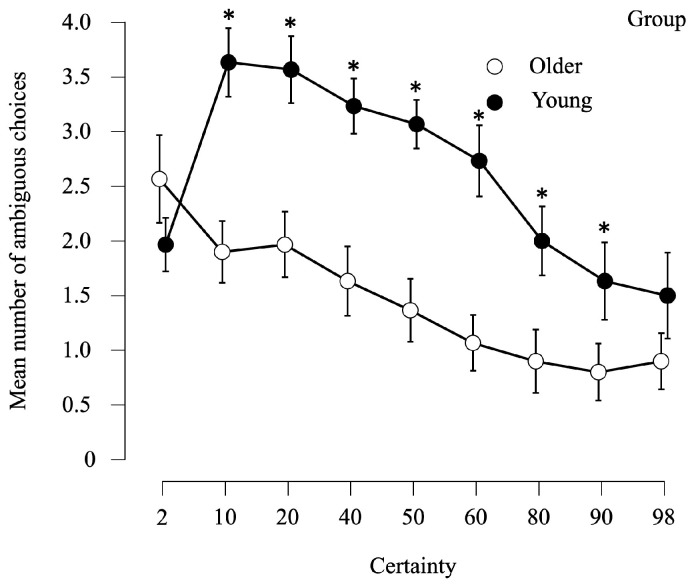
Average number of ambiguous options chosen in function of the certainty. The bars represent standard errors, with a maximum of 4 ambiguous options selectable per certainty level, expressed in percentages. The stars indicate significant comparisons, *p* < 0.001.

A repeated-measures ANOVA was performed to investigate the effect of the percentage of certainty on the number of ambiguous choices. A significant simple effect of percentage of certainty (F(1, 58) = 34.9, *p* < 0.001, η^2^_p_ = 0.375), a significant simple effect of group (F(1, 58) = 51.6, *p* < 0.001, η^2^_p_ = 0.469) and a significant percentage of certainty*group interaction effect (F(1, 58) = 13.6, *p* < 0.001, η^2^ = 0.191) were observed (Figure 5). The planned comparisons, with Bonferroni correction, showed that older adults selected significantly fewer ambiguous options than young adults for all percentages of certainty (all *p* < 0.001) except 2% and 98% of certainty.

Concerning the format of the numerical information (frequencies vs percentages), there was neither a significant effect of numerical presentation (F(1, 58) = 2.3, *p* = 0.133, η^2^ = 0.012) nor numerical presentation*group interaction effect (F(1, 58) = 2.2, *p* = 0.152, η^2^ = 0.011) (see Figure 4b). The effect of the group was significant.

### 3.2. Regression Analyses

Stepwise multiple linear regression analyses were performed for each score on the decision-making task (i.e., the total number of ambiguous choices and the total number of risky choices in the gain and loss contexts), with the scores of the episodic memory tasks being entered as explanatory variables in the following order: immediate d’ index R and K response, delayed d’ index recollection and familiarity response, immediate C index recollection and familiarity response, delayed C index recollection and familiarity response, immediate recall and delayed recall. We controlled the effect of age in each model by using the bootstrap method by percentiles, which is considered more robust than the correction of bias [63], and we performed 5000 replications for each model. Thus, 11 models were obtained. Our goal was to check whether episodic memory performances could significantly explain decision-making competence and whether this could be moderated by age. Our hypothesis was that the high scores in episodic memory tasks significantly explain the scores in decision-making tasks and do so to an even greater extent in young participants.

All the models for each decision-making score for a direct effect of age in the memory tests are presented in Table 2 and in Supplementary Material.

With regard to ambiguous choices, the standardised effect of immediate d’ index recollection responses was significant from model 10 onwards, i.e., in the presence of immediate and delayed recall, *β* = 0.32, t = 2.09, *p* = 0.04 (model 10) and *β* = 0.41, t = 2.59, *p* = 0.001 (model 11), respectively. The effect of age was significant for all models.

For the total score for risky choices, all the models were significant (see Supplementary Material). Despite this, none of the introduced variables had a significant effect on the model. The only exception was age, whose effect remained significant in all the models, from a minimum value of *β* = −0.43, t = −2.79, *p* = 0.009 (model 9) to a maximum value of *β* = −0.65, t = −5.28, *p* = 0.001 (model 1).

For risky choices in the gain context, the effect of age was significant up to model 3, *β* = −0.35, t = −2.35, *p* = 0.02 (model 1), *β* = −0.35, t = −2.29, *p* = 0.02 (model 2), *β* = −0.34, t = −2.26, *p* = 0.03 (model 3), and no other standardised coefficient was significant for any model (see Supplementary Material).

For risky choices in the loss context, all the models were significant, and the effect of age remained significant for all models except model 11, *β* = −0.35, t = −1.85, *p* = 0.07, while the immediate d’ index familiarity response was significant in model 5, *β* = 0.32, t = −1.99, *p* = 0.05 and model 7, *β* = 0.38, t = 2.23, *p* = 0.03. In model 7, the significance of the effect of delayed C index recollection responses was significant, *β* = 0.27, t = 1.96, *p* = 0.05, even though this was no longer significant in subsequent models (see Supplemental Material).

With regard to the effects of age, we verified the moderating influence of this factor on the significant effects mentioned above. Concerning the effect of immediate d’ index recollection response type on the ambiguous choices, age had no moderating effect, whatever its value. The absence of any moderating effect of age was also observed for the effect of the immediate d’ index familiarity responses type on the risky choices in the loss context and for the effect of the delayed C index recollection responses type on risky choices in the loss context. These results are summarised in Table 3 and Table 4.

## 4. Discussion

### 4.1. The Effect of Age on Episodic Memory

The recall task yielded two key observations: (1) recall abilities declined with ageing, and (2) this decline was observed both immediately and after three-and-a-half hours. This pattern of results is consistent with the literature [39,64,65,66,67] and can depend on some specific traits (see [32,33]). Since recall is cognitively demanding, it appears particularly vulnerable to the decrease in cognitive resources with age [65]. It was proposed that the age-related decline in recall may partly be due to less effective self-initiated encoding or retrieval strategies in older adults as compared to younger adults [39,66]. Even though our study used a deep encoding strategy, older adults’ performance remained inferior to that of young adults, suggesting that encouraging older adults to use such strategies may not fully overcome their recall difficulties. Furthermore, it should be noted that, in the present study, the recall tasks, both immediate and delayed, were always performed after the recognition task, so the results and their interpretation, while consistent with the current literature, must be taken with caution.

Concerning the recognition performances, the ability to distinguish between old and new items (i.e., d’ index) was again better in the young than in the older adults, and this was for immediate and delayed recognition but only for familiarity response type. The difference was not observed for recollection response type. These results are somewhat surprising, as most studies using recognition tasks that measure recollection and familiarity generally report a decline in recollection and preservation of the familiarity process with age [40,43]. However, our results are similar to those of [45], who reported that older adults with high memory performance showed intact recollection and impaired familiarity as compared to young adults, while the reverse was true for older adults with lower memory performance. One possible explanation for our results could be the high educational level of our older adult sample, as higher education is associated with better episodic memory performance [68,69]. Moreover, the deep encoding strategy may have increased recollection responses in older adults while reducing familiarity responses. Given that older adults often have difficulty implementing self-initiated strategies, our task, which provided a strategy during encoding (i.e., self or celebrity comparison), may counteract this difficulty. In addition, the response strategy (i.e., C index) showed that both groups exhibited a relative liberal response bias (i.e., they often accepted items as having been previously seen), although this tendency was less pronounced in the older group for familiarity-based responses. This result is partly consistent with that of [70], who described more conservative criteria in older adults, potentially due to their larger vocabulary, which could lead to variability in the distribution of the new items (i.e., relative to Signal Detection Theory), resulting in a more conservative strategy. This conservative approach in older adults could also contribute to the decrease in familiarity-based responses. Finally, the method we used to measure recollection and familiarity, which differs from the standard R/K paradigm, could have influenced the observed results.

### 4.2. The Effect of Age on Decision-Making

In the scenarios task, young adults sought significantly more risk and ambiguity than older adults, consistent with previous research [7,16,71]. Socioemotional Selectivity Theory (SST) may explain this phenomenon, with older adults’ decisions being guided by the maximisation of well-being and young adults’ decisions being influenced by the pursuit of knowledge [72,73,74,75]. Older adults would thus feel more secure in selecting the most likely options, while young adults would be curious about the less likely options.

More precisely, both groups used similar strategies in the under-risk condition, avoiding risk in the context of gain and favouring it in the context of loss. This framing effect (i.e., positive vs. negative) is well documented: people are more likely to secure a gain (i.e., avoiding risk) and to try to avoid a loss (i.e., taking risks) [76]. While both groups appeared to adapt their decisions to the context in a similar way, the percentage of certainty influenced their strategies differently. In the context of gain, older adults took more risks as the percentage of certainty increased (i.e., to achieve higher gains). The young adults, however, took more risks at 2%, 60% and 90% certainty levels. While older adults’ behaviour seems logical (i.e., maximise the chance of achieving a gain), young adults’ behaviour is less easy to understand. It is possible that a very low level of certainty (i.e., 2%) reinforced the novelty-seeking effect, encouraging young adults to take risks. However, it’s not clear why 60% and 90% certainty led to such risky decisions. In the context of loss, older adults took fewer risks overall as the percentage of certainty increased, probably with the aim of minimising the risk of loss, consistent with the importance they place on well-being. Young adults, conversely, tended to take more risks as the percentage of certainty increased. This behaviour is reminiscent of the phenomenon of self-perceived invincibility, which evolves over the course of life [77]. Young adults may perceive themselves as more invincible than older adults, leading them to select more riskier options.

In the under-ambiguity condition, which used only the context of gain, both groups used the same strategy; the higher the percentage of certainty, the fewer ambiguous options they chose. Our results are in line with the literature, which has described ambiguity aversion as moderate to high ambiguity in the general population, especially in gain context [78].

Finally, the numerical presentation format impacted decision-making under risk but not under ambiguity, with more risks being taken when information was given as a frequency rather than as a percentage. As decisions under risk are thought to rely more on the reflective system, and thus on ratio processing, than decisions under ambiguity [79,80], it seems understandable that decisions under risk are more influenced by numerical presentation than decisions under ambiguity. Moreover, as frequencies are known to be harder to process than percentages [81,82], the risk might be less well perceived, engendering more risk-taking when numerical values are presented in frequencies.

However, the differences we observed between young and older adults in decision-making under risk and under ambiguity must be considered with caution. In fact, the decision contexts in the two tasks that we designed cannot be simply considered completely equivalent, and other reasons relating to the participants’ decision-making under conditions of risk and ambiguity may, therefore, explain the observed age differences. In addition, the mood of the participants could have influenced their decision-making processes: on average, the older adults were in a more positive mood than the younger adults, but they were also more aroused.

### 4.3. The Impact of Episodic Memory on Decision-Making

In the present study, we assumed that because the recollection process is compromised with ageing, older adults may preferentially rely on familiarity-based cues to make decisions. This reliance on familiarity could lead to a preference for familiar, less uncertain and less risky options. A decline in the recollection process in older adults could, therefore, contribute to more conservative decision-making. However, it is important to mention before discussing the regression results that in the present study, the recollection process was preserved in older adults contrary to the familiarity process.

Our regression analyses showed direct effects (after controlling for age and testing for its moderating effect) of the immediate d’ index recollection response type on the choice of ambiguous options, and of the immediate d’ index familiarity response type and the delayed C index recollection responses type on the choice of risky options in the loss context. Thus, our data show that, to some extent, decision-making is influenced by episodic memory functioning. However, it seems that our hypothesis is not confirmed, given that recollection and familiarity processes had some effect on decision-making under risk and under ambiguity in both young and older adults. Both young and older adults mobilised their episodic memories to support their decision-making. [19] reported that both younger and older adults are capable of using episodic memory in decision-making. Nevertheless, they showed that younger adults seem to suppress semantic memory when making decisions if a relevant episodic memory is available. In contrast, older adults appear to rely on semantic memory regardless of the relevance of episodic memory. This suggests that older adults may have difficulty filtering out irrelevant information or that they may be less efficient in using episodic memory. Inhibitory control and updating processes might play a role in how older adults prioritise competing memory sources during decision-making [7].

Studies investigating the impact of precise memories on decision-making [23,83] have suggested that recollection influences decision-making. Nevertheless, their findings are difficult to compare with ours due to differences in protocol and the fact that they did not consider the impact of familiarity.

It was proposed that people typically rely on processes that combine the greatest efficiency with the lowest cognitive cost [84], even during complex processing like decision-making. Familiarity is thought to be less cognitively demanding than recollection [43], so it might be preferred in decision-making situations. Moreover, relying on familiar impressions rather than recalling specific memories may be a more generalisable strategy that can guide a wider range of decisions. However, our study seems to show that both familiarity and recollection can be used when decision-making involves episodic memory, depending on whether the decision is made under ambiguity or under risk.

## 5. Conclusions

This study found lower recall and recognition performances with age. However, it also observed that deep encoding promoted recollection at retrieval and was associated with comparable numbers of recollection-type hits and similar d’ index in young and older adults. When performing the scenarios task, which is supposed to be a good reflection of everyday life decisions, young adults always sought more risk and ambiguity than their elder counterparts in the under-risk and under-ambiguity conditions, respectively. Socioemotional Selectivity Theory provides an interesting explanation for these opposed tendencies. Young adults’ decision-making would be driven by a need to feed their curiosity and older adults’ decision-making would be driven by a need to enhance their well-being. Finally, in both young and older adults, the processes of recollection and familiarity could partly explain performances in decision-making tasks under ambiguity and under risk, respectively.

## Figures and Tables

**Table 1:** Scores (mean and SD) of young and older adults on the episodic memory task, the value of a statistical test (*t*-test or U-test), *p*-value and effect size (Cohen’s d for *t*-test, rank biserial correlation for Mann–Whitney test).

	Young Adults Mean (SD)	Older Adults Mean (SD)	Test	*p*, Effect Size
**Recall**				
Immediate recall	10.9 (4.2)	6.2 (3.3)	U(56) = 146.5	<0.001, −0.65
Delayed recall	9.4 (4.0)	5.8 (3.6)	U(58) = 198.0	<0.001, −0.56
**d’ index recollection-type**				
Immediate recognition	0.59 (0.54)	0.88 (0.71)	t(55) = 1.71	0.092, 0.45
Delayed recognition	−0.05 (0.49)	0.19 (0.56)	t(51) = 1.70	0.095, 0.46
**d’ index familiarity-type**				
Immediate recognition	1.15 (0.48)	0.32 (0.43)	t(52) = −6.57	<0.001, −1.80
Delayed recognition	0.77 (0.42)	0.29 (0.48)	t(49) = −3.80	<0.001, −1.07
**C index recollection-type**				
Immediate recognition	0.71 (0.28)	0.77 (0.21)	U(55) = 437.0	0.605, 0.08
Delayed recognition	0.90 (0.30)	0.94 (0.31)	t(51) = 0.43	0.667, 0.11
**C index familiarity-type**				
Immediate recognition	0.98 (0.28)	1.37 (0.29)	U(52) = 604	<0.001, 1.38
Delayed recognition	1.08 (0.41)	1.35 (0.30)	t(49) = −3.80	<0.02, 0.74
**Hits recollection-type**				
Immediate recognition	8.80 (4.12)	9.80 (3.78)	t(58) = 0.97	0.332, 0.25
Delayed recognition	4.80 (3.17)	5.80 (3.05)	U(58) = 532	0.222, 0.18
**Hits familiarity-type**				
Immediate recognition	9.53 (3.77)	3.56 (2.50)	U(58) = 70.5	<0.001, −0.84
Delayed recognition	7.16 (4.06)	3.23 (2.67)	U(58) = 165.5	<0.001, −0.63
**FA Recollection-type**				
Immediate recognition	3.1 (2.0)	2.1 (2.4)	U(58) = 291	0.017, −0.35
Delayed recognition	3.5 (2.3)	3.1 (2.5)	U(58) = 394	0.407, −0.12
**FA Familiarity-type**				
Immediate recognition	0.6 (1.0)	0.7 (0.8)	U(58) = 491	0.495, 0.09
Delayed recognition	1.0 (1.8)	0.7 (1.3)	U(58) = 422	0.638, −0.06

**Table 2:** Regression analyses with effect sizes and significance of models.

	*n*	*R* ^2^ *_adj_*	*F*	*p*	Δ*R*^2^*_adj_*	*p*
Ambiguous total	60					
Model 1		0.23	13.00	0.001	-	-
Model 2		0.26	7.97	0.001	0.05	0.12
Model 3		0.25	5.51	0.003	0.01	0.41
Model 4		0.23	4.03	0.008	0.001	0.90
Model 5		0.22	3.23	0.16	0.01	0.48
Model 6		0.20	2.66	0.03	0.001	0.94
Model 7		0.18	2.23	0.56	0.002	0.75
Model 8		0.17	2.00	0.79	0.01	0.46
Model 9		0.19	2.03	0.07	0.03	0.19
Model 10		0.24	2.23	0.04	0.06	0.09
Model 11		0.29	2.45	0.03	0.06	0.09
Risky total	58					
Model 1		0.40	27.90	<0.001	-	-
Model 2		0.39	13.65	<0.001	0.001	0.79
Model 3		0.37	8.98	<0.001	0.003	0.64
Model 4		0.42	8.09	<0.001	0.05	0.07
Model 5		0.40	6.31	<0.001	0.001	0.83
Model 6		0.38	5.12	<0.001	0.001	0.85
Model 7		0.39	4.57	<0.001	0.02	0.29
Model 8		0.42	4.62	<0.001	0.04	0.09
Model 9		0.43	4.41	<0.001	0.03	0.19
Model 10		0.42	3.87	0.002	0.002	0.80
Model 11		0.41	3.49	0.003	0.006	0.54
Risky gain	60					
Model 1		0.10	5.51	0.02	-	-
Model 2		0.08	2.82	0.07	0.005	0.62
Model 3		0.06	1.84	0.16	0.001	0.89
Model 4		0.04	1.39	0.26	0.003	0.69
Model 5		0.02	1.13	0.37	0.004	0.66
Model 6		−0.01	0.92	0.49	0.001	0.79
Model 7		−0.04	0.80	0.59	0.005	0.65
Model 8		0.01	1.05	0.42	0.06	0.12
Model 9		−0.007	0.97	0.49	0.01	0.52
Model 10		−0.04	0.85	0.59	0.001	0.89
Model 11		−0.05	0.74	0.61	0.02	0.38
Risky loss	60					
Model 1		0.27	16.03	<0.001	-	-
Model 2		0.26	8.32	0.001	0.01	0.40
Model 3		0.26	5.64	0.003	0.009	0.48
Model 4		0.31	5.43	0.002	0.06	0.06
Model 5		0.30	4.41	0.003	0.01	0.45
Model 6		0.30	3.57	0.007	0.001	0.96
Model 7		0.28	3.87	0.004	0.06	0.06
Model 8		0.33	3.31	0.007	0.001	0.74
Model 9		0.32	3.01	0.011	0.01	0.38
Model 10		0.29	2.64	0.012	0.001	0.75
Model 11		0.27	2.33	0.034	0.001	0.80

**Table 3:** Moderating effects of age.

	β	*p*
Ambiguous (Model 1)		
immediate d’ index recollection response	1.09	0.27
Age	−0.09	<0.001
immediate d’ index recollection response *Age	−0.05	0.26
Risky loss (Model 2)		
Immediate d’ index familiarity response	0.13	0.88
Age	−0.04	0.03
Immediate d’ index familiarity response *Age	0.003	0.93
Risky loss (Model 3)		
immediate d’ index recollection response	0.13	0.87
Age	−0.04	0.04
immediate d’ index recollection response *Age	0.003	0.93

**Table 4:** Marginal estimated means.

	Mean (SE)	*p*
Model 1		
Mean Age	1.08 (0.98)	0.27
Young (−1 ET)	2.29 (1.53)	0.15
Older (+1 ET)	−0.12 (1.40)	0.92
Model 2		
Mean age	0.13 (0.89)	0.89
Young (−1 ET)	0.05 (1.15)	0.97
Older (+1 ET)	0.20 (0.91)	0.83
Model 3		
Mean age	0.12 (0.87)	0.87
Young (−1 ET)	0.05 (1.51)	0.97
Older (+1 ET)	0.20 (0.92)	0.83

## Data Availability

The research materials and data are available on the Open Science Framework at https://osf.io/j4wzr/?view_only=6ad2e97e00c74e9991d26cda97fe7330 (accessed on 2 September 2024).

## Supplementary Materials

The following supporting information can be downloaded at: https://www.mdpi.com/article/10.3390/brainsci14100998/s1, Table S1: Regression analysis. Direct effects.
