# Supplementary material for: The Impact of Episodic Memory on Decision-Making in Aging: Scenarios from Everyday Life Situations
Source: Brain Sci. 2024 Sep 30;14(10):998. doi: 10.3390/brainsci14100998 (PMC11506795; doi:10.3390/brainsci14100998)
Supplement: Supplementary file 1 [file brainsci-14-00998-s001.zip › brainsci-3214635-supplementary.pdf]

	β	t	p
**Ambiguous total**			
Model 1			
Age	-0,50	-3,61	<0,001
Model 2			
Age	-0,51	-3,74	<0,001
immediate d′ index R response	0,21	1,57	0,13
Model 3			
Age	-0,50	-3,67	<0,001
immediate d′ index R response	0,25	1,73	0,09
delayed d′ index R response	-0,12	-0,84	0,41
Model 4			
Age	0,49	-2,99	0,005
immediate d′ index R response	0,25	1,71	0,09
delayed d′ index R response	-0,12	-0,83	0,41
immediate d′ index K response	0,02	0,12	0,91
Model 5			
Age	-0,52	-3,07	0,004
immediate d′ index R response	0,26	1,69	0,10
delayed d′ index R response	-0,14	-0,94	0,35
immediate d′ index K response	0,04	0,25	0,81
delayed d′ index K response	-0,11	-0,72	0,48
Model 6			
Age	-0,53	-2,29	0,006
immediate d′ index R response	0,25	1,66	0,11
delayed d′ index R response	-0,14	-0,93	0,36
immediate d′ index K response	0,04	0,20	0,84
delayed d′ index K response	-0,12	-0,71	0,48
immediate C index R response	-0,01	-0,07	0,94
Model 7			
Age	0,52	-2,85	0,007
immediate d′ index R response	0,24	1,63	0,11
delayed d′ index R response	-0,14	-0,91	0,37
immediate d′ index K response	0,05	0,25	0,80
delayed d′ index K response	-0,12	-0,72	0,48
immediate C index R response	-0,03	-0,13	0,90
delayed C index R response	0,05	0,33	0,75
Model 8			
Age	-0,49	-2,65	0,01
immediate d′ index R response	0,25	1,68	0,10
delayed d′ index R response	-0,13	-0,86	0,39
immediate d′ index K response	0,04	-0,17	0,87
delayed d′ index K response	-0,09	-0,60	0,56
immediate C index R response	-0,01	-0,08	0,93
delayed C index R response	0,03	0,17	0,87
immediate C index K response	-0,15	-0,75	0,46
Model 9			
Age	-0,47	-2,55	0,02
immediate d′ index R response	0,23	1,53	0,14
delayed d′ index R response	-0,12	-0,80	0,43
immediate d′ index K response	-0,04	-0,19	0,85
delayed d′ index K response	-0,02	-0,11	0,92
immediate C index R response	-0,02	-0,13	0,89
delayed C index R response	0,01	0,02	0,98
immediate C index K response	-0,25	-1,22	0,23
delayed C index K response	0,24	1,36	0,19
Model 10			
Age	-0,55	-2,98	0,006
immediate d′ index R response	0,32	2,09	0,05
delayed d′ index R response	-0,10	-0,70	0,49
immediate d′ index K response	0,07	0,32	0,74
delayed d′ index K response	0,03	0,20	0,84

immediate C index R response	-0,03	-0,17	0,87
delayed C index R response	0,03	0,19	0,85
immediate C index K response	-0,30	-1,51	0,14
delayed C index K response	0,24	1,42	0,17
immediate recall	-0,34	-1,71	0,09
Model 11			
Age	-0,66	-3,49	0,002
immediate d' index R response	0,41	2,59	0,01
delayed d' index R response	-0,06	-0,43	0,76
immediate d' index K response	-0,01	-0,03	0,99
delayed d' index K response	0,14	0,78	0,44
immediate C index R response	-0,12	-0,72	0,48
delayed C index R response	0,06	0,38	0,71
immediate C index K response	-0,31	-1,53	0,14
delayed C index K response	0,21	1,28	0,21
immediate recall	-0,19	-0,09	0,37
delayed recall	-0,38	-1,76	0,09
**Risky total**			
Model 1			
Age	-0,65	-5,28	<0,001
Model 2			
Age	-0,65	-5,23	<0,01
immediate d' index R response	0,03	0,26	0,80
Model 3			
Age	-0,65	-5,19	<0,01
immediate d' index R response	0,01	0,12	0,90
delayed d' index R response	0,06	0,46	0,65
Model 4			
Age	-0,51	-3,52	0,001
immediate d' index R response	0,03	0,24	0,82
delayed d' index R response	0,06	0,51	0,61
immediate d' index K response	0,28	1,88	0,07
Model 5			
Age	-0,51	-3,42	0,002
immediate d' index R response	0,03	0,23	0,82
delayed d' index R response	0,06	0,46	0,65
immediate d' index K response	0,28	1,87	0,07
delayed d' index K response	-0,03	-0,21	0,83
Model 6			
Age	-0,52	-3,33	0,002
immediate d' index R response	0,03	0,23	0,82
delayed d' index R response	0,06	0,42	0,68
immediate d' index K response	0,27	1,64	0,11
delayed d' index K response	-0,03	-0,24	0,81
immediate C index R response	0,03	-0,19	0,85
Model 7			
Age	0,50	-3,18	0,003
immediate d' index R response	0,03	0,19	0,85
delayed d' index R response	0,06	0,42	0,68
immediate d' index K response	0,30	1,80	0,08
delayed d' index K response	-0,04	-0,29	0,77
immediate C index R response	-0,05	-0,04	0,71
delayed C index R response	0,14	1,08	0,29
Model 8			
Age	0,45	-2,89	0,007
immediate d' index R response	0,05	0,37	0,72
delayed d' index R response	0,07	0,53	0,60
immediate d' index K response	0,13	0,70	0,49
delayed d' index K response	-0,006	-0,05	0,96
immediate C index R response	-0,04	-0,29	0,77
delayed C index R response	0,10	0,74	0,47
immediate C index K response	-0,28	-1,74	0,09
Model 9			
Age	-0,42	-2,79	0,009

immediate d' index R response	0,03	0,21	0,84
delayed d' index R response	0,08	0,60	0,55
immediate d' index K response	0,13	0,69	0,50
delayed d' index K response	0,06	0,41	0,69
immediate C index R response	-0,04	-0,34	0,73
delayed C index R response	0,08	0,60	0,55
immediate C index K response	-0,37	-2,14	0,04
delayed C index K response	0,20	1,33	0,19
Model 10			
Age	-0,45	-2,76	0,01
immediate d' index R response	0,05	0,33	0,74
delayed d' index R response	0,08	0,62	0,54
immediate d' index K response	0,15	0,76	0,45
delayed d' index K response	0,07	0,47	0,65
immediate C index R response	-0,04	-0,35	0,73
delayed C index R response	0,08	0,62	0,54
immediate C index K response	-0,38	-2,14	0,04
delayed C index K response	0,20	1,32	0,20
immediate recall	-0,07	-0,39	0,70
Model 11			
Age	-0,48	-2,79	0,009
immediate d' index R response	0,07	0,51	0,62
delayed d' index R response	0,09	0,71	0,48
immediate d' index K response	0,13	0,62	0,54
delayed d' index K response	0,10	0,64	0,53
immediate C index R response	-0,08	-0,53	0,61
delayed C index R response	0,09	0,68	0,50
immediate C index K response	-0,38	-2,11	0,04
delayed C index K response	0,19	1,23	0,23
immediate recall	-0,02	-0,10	0,93
delayed recall	-0,13	-0,63	0,54
**Risky gain**			
Model 1			
Age	-0,35	-2,35	0,02
Model 2			
Age	-0,35	-2,29	0,02
immediate d' index R response	-0,07	-0,49	0,62
Model 3			
Age	-0,34	-2,26	0,03
immediate d' index R response	-0,07	-0,43	0,67
delayed d' index R response	-0,02	-0,13	0,90
Model 4			
Age	-0,31	-1,67	0,10
immediate d' index R response	-0,07	-0,40	0,69
delayed d' index R response	-0,02	-0,12	0,90
immediate d' index K response	0,07	0,40	0,69
Model 5			
Age	-0,29	-1,49	0,14
immediate d' index R response	-0,06	-0,39	0,70
delayed d' index R response	-0,01	-0,04	0,97
immediate d' index K response	0,05	0,30	0,76
delayed d' index K response	0,08	0,44	0,66
Model 6			
Age	-0,29	-1,49	0,15
immediate d' index R response	-0,06	-0,37	0,71
delayed d' index R response	-0,01	-0,07	0,94
immediate d' index K response	0,04	0,18	0,86
delayed d' index K response	0,07	0,38	0,70
immediate C index R response	-0,05	-0,26	0,80
Model 7			
Age	-0,32	-1,52	0,14
immediate d' index R response	-0,06	-0,35	0,73
delayed d' index R response	-0,01	-0,07	0,94
immediate d' index K response	0,02	0,10	0,92

delayed d′ index K response	0,07	0,40	0,69
immediate C index R response	-0,03	-0,17	0,87
delayed C index R response	-0,08	-0,46	0,65
Model 8			
Age	-0,25	-1,23	0,23
immediate d′ index R response	-0,03	-0,21	0,84
delayed d′ index R response	0,02	0,02	0,99
immediate d′ index K response	-0,18	-0,73	0,47
delayed d′ index K response	0,12	0,64	0,53
immediate C index R response	-0,02	-0,08	0,93
delayed C index R response	-0,13	-0,78	0,44
immediate C index K response	0,34	-1,59	0,12
Model 9			
Age	-0,24	-1,16	0,26
immediate d′ index R response	-0,05	-0,28	0,78
delayed d′ index R response	0,01	0,05	0,96
immediate d′ index K response	-0,18	-0,73	0,45
delayed d′ index K response	0,16	0,81	0,42
immediate C index R response	-0,02	-0,11	0,91
delayed C index R response	-0,14	-0,85	0,41
immediate C index K response	-0,40	-1,70	0,10
delayed C index K response	0,13	0,65	0,52
Model 10			
Age	-0,25	-1,14	0,27
immediate d′ index R response	-0,04	-0,21	0,84
delayed d′ index R response	0,01	0,06	0,95
immediate d′ index K response	-0,17	-0,65	0,52
delayed d′ index K response	0,16	0,81	0,42
immediate C index R response	-0,02	-0,11	0,91
delayed C index R response	-0,14	-0,81	0,42
immediate C index K response	-0,40	-1,68	0,10
delayed C index K response	0,13	0,64	0,53
immediate recall	-0,03	-0,14	0,89
Model 11			
Age	-0,31	-1,36	0,18
immediate d′ index R response	0,01	0,07	0,94
delayed d′ index R response	0,04	0,20	0,84
immediate d′ index K response	-0,21	-0,80	0,43
delayed d′ index K response	0,23	1,06	0,30
immediate C index R response	-0,07	-0,39	0,70
delayed C index R response	-0,12	-0,71	0,48
immediate C index K response	-0,40	-1,66	0,11
delayed C index K response	0,11	0,54	0,59
immediate recall	0,06	0,23	0,82
delayed recall	-0,24	-0,89	0,38
**Risky loss**			
Model 1			
Age	-0,05	-4,00	<0,001
Model 2			
Age	-0,55	-4,03	<0,001
immediate d′ index R response	0,12	0,85	0,40
Model 3			
Age	-0,55	-4,04	<0,001
immediate d′ index R response	0,09	0,62	0,54
delayed d′ index R response	0,10	0,71	0,48
Model 4			
Age	-0,39	-2,51	0,02
immediate d′ index R response	0,10	0,75	0,46
delayed d′ index R response	0,11	0,77	0,45
immediate d′ index K response	0,30	1,89	0,07
Model 5			
Age	-0,42	-2,61	0,01
immediate d′ index R response	0,10	0,72	0,47
delayed d′ index R response	0,09	0,62	0,54

immediate d' index K response	0,32	1,99	0,05
delayed d' index K response	-0,12	-0,77	0,45
Model 6			
Age	-0,42	-2,49	0,02
immediate d' index R response	0,09	0,71	0,48
delayed d' index R response	0,09	0,61	0,55
immediate d' index K response	0,32	1,85	0,07
delayed d' index K response	-0,11	-0,74	0,47
immediate C index R response	0,01	0,06	0,95
Model 7			
Age	-0,38	-2,33	0,03
immediate d' index R response	0,09	0,67	0,51
delayed d' index R response	0,09	0,63	0,53
immediate d' index K response	0,38	2,23	0,03
delayed d' index K response	-0,13	-0,86	0,40
immediate C index R response	-0,04	-0,29	0,78
delayed C index R response	0,27	1,96	0,05
Model 8			
Age	-0,37	-2,19	0,04
immediate d' index R response	0,10	0,69	0,49
delayed d' index R response	0,09	0,64	0,53
immediate d' index K response	0,35	1,71	0,09
delayed d' index K response	-0,12	-0,79	0,44
immediate C index R response	-0,03	-0,26	0,79
delayed C index R response	0,26	1,82	0,08
immediate C index K response	-0,06	-0,34	0,74
Model 9			
Age	-0,36	-2,09	0,04
immediate d' index R response	0,08	0,58	0,57
delayed d' index R response	0,10	0,69	0,50
immediate d' index K response	0,34	1,69	0,10
delayed d' index K response	-0,07	-0,44	0,66
immediate C index R response	-0,04	-0,30	0,77
delayed C index R response	0,24	1,71	0,09
immediate C index K response	-0,13	-0,66	0,52
delayed C index K response	0,14	0,89	0,38
Model 10			
Age	-0,37	-2,08	0,04
immediate d' index R response	0,09	0,65	0,52
delayed d' index R response	0,10	0,70	0,49
immediate d' index K response	0,36	1,69	0,10
delayed d' index K response	-0,06	-0,37	0,72
immediate C index R response	-0,05	-0,30	0,77
delayed C index R response	0,25	1,71	0,10
immediate C index K response	-0,14	-0,69	0,50
delayed C index K response	0,15	0,88	0,39
immediate recall	-0,06	-0,32	0,75
Model 11			
Age	-0,35	-1,85	0,07
immediate d' index R response	0,09	0,53	0,60
delayed d' index R response	0,09	0,64	0,53
immediate d' index K response	0,37	1,68	0,10
delayed d' index K response	-0,08	-0,43	0,67
immediate C index R response	-0,03	-0,20	0,84
delayed C index R response	0,24	1,65	0,11
immediate C index K response	-0,14	-0,68	0,50
delayed C index K response	0,15	0,89	0,38
immediate recall	-0,08	-0,39	0,70
delayed recall	0,06	0,25	0,80

Table S1: Regression analysis. Direct effects.
